# Total Syntheses of Telisatin A, Telisatin B and Lettowianthine

**DOI:** 10.3390/molecules14030917

**Published:** 2009-02-26

**Authors:** Surachai Nimgirawath, Phansuang Udomputtimekakul

**Affiliations:** Department of Chemistry, Faculty of Science, Silpakorn University; Nakorn Pathom 73000, Thailand; E-mail: Phansuang@yahoo.com (P.U.)

**Keywords:** Alkaloid, Dioxoaporphine, Isoquinoline, Radical cyclisation, Synthesis

## Abstract

Treatment of 1-(2-bromoarylmethyl)-3,4-dihydroisoquinolines with oxalyl chloride and triethylamine gave 1-(2-bromophenyl)-5,6-dihydropyrrolo[2,1-a]isoquinoline-2,3-dione derivatives, for example, 1-(2-bromophenyl)-5,6-dihydro-8,9-dimethoxypyrrolo[2,1-a]isoquinoline-2,3-dione**.** Radical cyclisation of these derivatives with tributyltin hydride and 1,1ʹ-azobis(cyclohexanecarbonitrile) afforded telisatin A, telisatin B and lettowianthine.

## Introduction

The telisatin-type aporphine alkaloids form a very small sub-group of the aporphine alkaloids in which N-6 and C-7 are fused to an oxalyl function. To date only five members of this type of aporphine alkaloids have been found to occur in Nature. These are telisatin A (**1**) and telisatin B (**2**) from *Telitoxicum peruvianum* Moldenke (Menispermaceae) [1], lettowianthine (**3**) and 11-methoxy-lettowianthine (**4**) from *Lettowianthus stellatus* Diels (Annonaceae) [2], and laurodionine (**5**) from *Phoebe formosana* Hayata (Lauraceae) [3]. Annonbraine, isolated from *Annona glaba* L (Annonaceae), was also assigned the same structure as lettowianthine (**3**), although there was a big difference in the melting points of the two alkaloids [4]. The structure of telisatin A was elucidated by comparison of spectral data and physical properties with a synthetic compound obtained by Saa and Cava [5], Castedo *et al.* [6] and Saa *et al*. [7]. The structures of the remaining alkaloids were assigned based on spectral data analysis.

**Figure 1:**
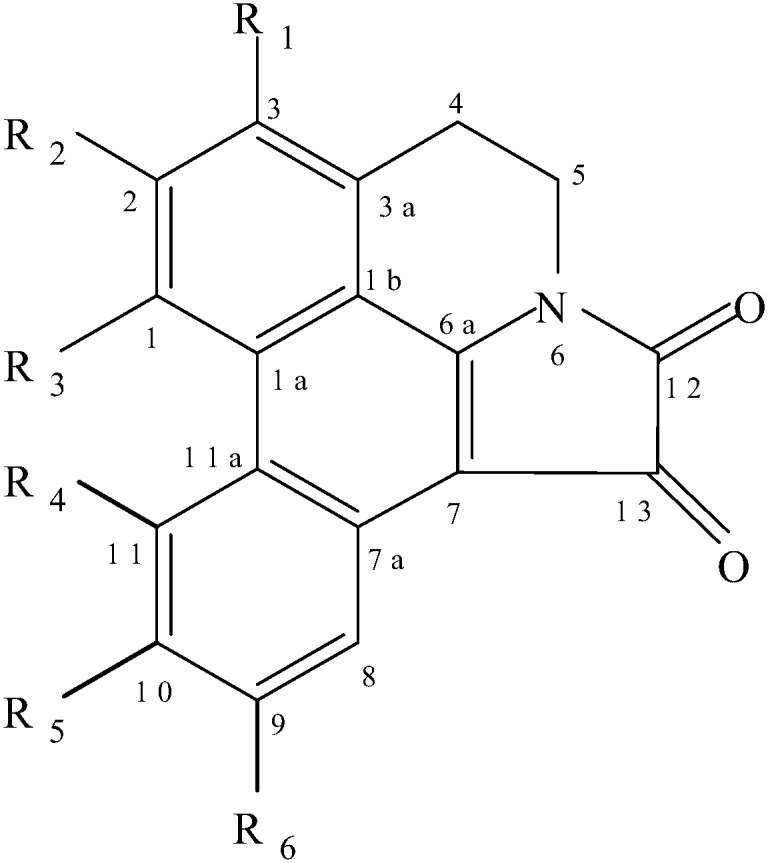
Structures of telisatin A (**1**), telisatin B (**2**), lettowianthine (annonbraine) (**3**), 11-methoxylettowianthine (**4**) and laurodionine (**5**).

1 : R_1_ = R_4_ = R_5_ = R_6_ = H, R_2_ = R_3_ = OCH_3_ (telisatin A)
2 : R_1_ = R_2_ = R_3_ = OCH_3_, R_4_ = R_5_ =R_6_ = H (telisatin B)
3 : R_1_ = R_4_ = R_5_ = R_6_ = H, R_2_ + R_3_ = OCH_2_O (lettowianthine, annonbraine)
4 : R_1_ = R_5_ = R_6_ = H, R_2_ + R_3_ = OCH_2_O, R_4_ = OCH_3_ (11-methoxylettowianthine)
5 : R_1_ = R_4_ = H, R_2_ = R_6_ = OH, R_3_ = R_5_ = OCH_3_ (laurodionine)

At present there are only two total syntheses of telisatin A reported by Castedo *et al*. [6] and by Saa *et al*. [7] The first method involved photochemical cyclisation in reasonably good yield (60%) of a very dilute (0.001 M) solution of 1-(2-bromophenyl)-5,6-dihydro-8,9-dimethoxypyrrolo[2,1-a]isoquinoline-2,3-dione (**10a**) [6]. This method has obvious inherent limitations with regards to its scalability and convenience. The second method, based on benzyne cycloaddition, gave a low yield (10%) [7]. A partial synthesis reported by Saa and Cava involved acylation of 6a,7-dehydronuciferine with oxalyl chloride [5]. Since 6a,7-dehydroaporphines themselves are not readily accessible, this method therefore lacks generality and convenience.

## Results and Discussion

**Scheme 1:**
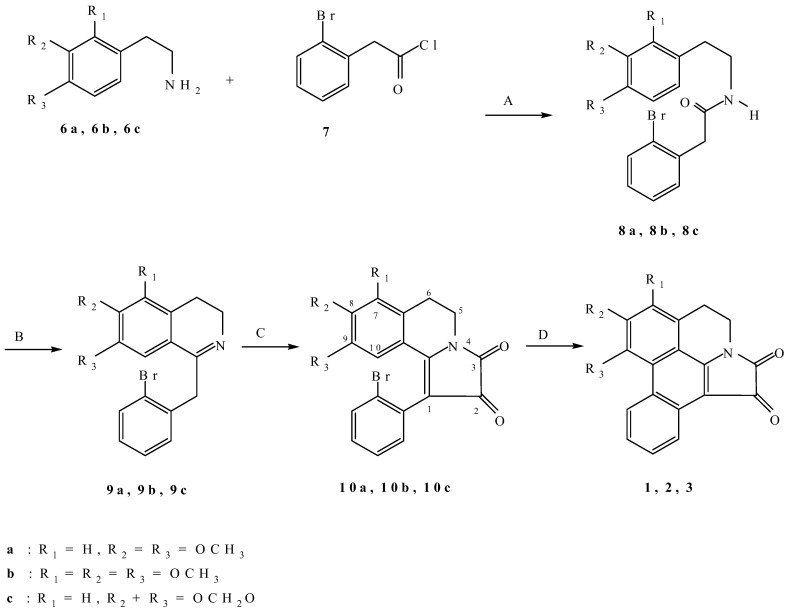
Syntheses of telisatin A (**1**), telisatin B (**2**) and lettowianthine (**3**).

We would like to report herein an extension of the first method described by Castedo *et al*. [6], shown in Scheme 1. Amides **8a**-**8c**, obtained by conventional methods, were converted by a Bischler–Napieralski reaction to dihydroisoquinolines **9a**-**9c**. We found that 1-(2-bromophenyl)-5,6-dihydropyrrolo[2,1-a]-isoquinoline-2,3-dione derivatives **10a**-**10c** could be more conveniently prepared by the reaction of dihydroisoquinolines **9a**-**9c** with oxalyl chloride in the presence of triethylamine with straightforward workup [8]. The antiplatelet activity of such 1-aryl-5,6-dihydropyrrolo[2,1-a]-isoquinoline-2,3-dione derivatives has been reported [9]. To overcome the limitations of photochemical cyclisation under extreme dilution and based on previous reports on the radical cyclisation of halostilbenes to phenanthrenes [10,11,12,13,14], solutions (0.025 M) of 1-(2-bromophenyl)-5,6-dihydropyrrolo[2,1-a]isoquinoline-2,3-dione derivatives **10a**-**10c** were treated with tributyltin hydride in the presence of 1,1ʹ-azobis(cyclohexanecarbonitrile) (ACCN) to give the corresponding telisatin-type alkaloids in 30-34% yields. 

*Reaction conditions*: A) 10 % NaHCO_3_/ chloroform; B) POCl_3_/ benzene; C) oxalyl chloride, triethylamine/ chloroform; D) Bu_3_SnH, ACCN/ dry toluene.

Fortunately, in all cases, it was possible to isolate the pure alkaloids from the crude reaction mixtures by simple crystallization from ethanol. Silica gel chromatography of the residues from the filtrates afforded only minute quantities of the pure alkaloids and was therefore not pursued any further. Comparison of the yields from radical cyclisation using ACCN with those obtained using 2,2ʹ-azobis(isobutyronitrile)(AIBN) was not possible since AIBN is no longer commercially available in Thailand. The spectral data of synthetic telisatin A (**1**), telisatin B (**2**) and lettowianthine (**3**) were in good agreement to those reported for the natural alkaloids.

## Conclusions

We have developed an easy and convenient synthesis of the telisatin-type alkaloids. Further applications of the current synthesis to the remaining telisatin-type alkaloids are in progress.

## Experimental

### General

Melting points were determined on a Stuart Scientific SMP 2 melting point apparatus and are uncorrected. Infrared spectra were recorded on CH_2_Cl_2_-films with a Perkin Elmer Spectrum GX FT-IR spectrophotometer. Ultraviolet spectra were recorded on methanol solutions with a Hitachi U-3300 spectrophotometer. ^1^H- and ^13^C-NMR spectra were recorded for deuterochloroform solutions at 300 MHz for ^1^H and 75 MHz for ^13^C with a Bruker AVANCE 300 spectrometer. Tetramethylsilane was used as the internal standard. High Resolution Mass spectra were recorded with a Bruker Daltonics MicrOTOF mass spectrometer. 

*N-(3,4-Dimethoxyphenethyl)-2-(2-bromophenyl)acetamide* (**8a**). A mixture of 2-bromophenylacetic acid (15.0 g, 0.07 mol) and thionyl chloride (20.8 g) in benzene (50 mL) was refluxed for 1 h. Removal of the solvent under vacuum gave 2-bromophenylacetyl chloride (**7**) which was dissolved in ethanol-free chloroform (50 mL) and added to a mixture of 3,4-dimethoxyphenethylamine (**6a**, 12.7 g, 0.07 mol) in chloroform (100 mL) and 10% sodium hydrogen carbonate (100 mL). The mixture was then stirred for 3 h and the chloroform layer was washed with water (2 × 100 mL), 10% hydrochloric acid (3 × 50 mL), water (100 mL), then dried over anhydrous sodium sulfate. Removal of the solvent under vacuum gave a residue which was recrystallized from ethanol to give amide **8a** as a pale yellow solid (22.0 g, 88.1%); m.p. 131-132 °C (Lit. [15] m.p. 127-129 °C); ^1^H-NMR: δ 7.54 (1H, d, *J* = 7.8 Hz, Ar-H); 7.30-7.22 (2H, m, Ar-H); 7.18-7.09 (1H, m, Ar-H); 6.72 (1H, d, *J* = 8.1 Hz, Ar-H); 6.63 (1H, d, *J* = 1.9 Hz, Ar-H); 6.60 (1H, dd, *J* = 8.1, 1.9 Hz, Ar-H); 5.58 (1H, s, NH); 3.85 (3H, s, OCH_3_); 3.82 (3H, s, OCH_3_); 3.66 (2H, s, CH_2_); 3.47 (2H, apparent q, *J* = 6.8 Hz, CH_2_); 2.71 (2H, apparent t, *J* = 7.0 Hz, CH_2_); ^13^C-NMR: δ 169.48(C), 148.98(C), 147.60(C), 134.79(C), 133.03(CH), 131.63(CH), 131.10(C), 129.04(CH), 127.92(CH), 124.94(C), 120.58(CH), 111.79(CH), 111.32(CH), 55.91(OCH_3_), 55.82 (OCH_3_), 43.99(CH_2_), 40.81(CH_2_), 34.99(CH_2_).

*N-(2,3,4-Trimethoxyphenethyl)-2-(2-bromophenyl)acetamide* (**8b**). In a similar manner, **8b** was obtained in 78.8% yield as a pale yellow solid from ethanol; m.p. 92-93 °C; ^1^H-NMR: δ 7.57 (1H, d, *J* = 8.3 Hz, Ar-H); 7.33-7.25 (2H, m, Ar-H); 7.19-7.12 (1H, m, Ar-H); 6.72 (1H, d, *J* = 8.5 Hz, Ar-H); 6.54 (1H, d, *J* = 8.5 Hz, Ar-H); 5.83 (1H, br s, NH); 3.88 (3H, s, OCH_3_); 3.83 (3H, s, OCH_3_); 3.79 (3H, s, OCH_3_); 3.67 (2H, s, CH_2_); 3.43 (2H, apparent q, *J* = 6.4 Hz, CH_2_); 2.70 (2H, apparent t, *J* = 6.7 Hz, CH_2_); ^13^C-NMR: δ 169.58(C), 152.59(C), 151.74(C), 142.19(C), 134.91(C), 133.05(CH), 131.78(CH), 128.97(CH), 127.89(CH), 125.10(C), 124.69(C), 124.46(CH), 107.42(CH), 60.92(OCH_3_), 60.75(OCH_3_), 56.01(OCH_3_), 44.05 (CH_2_), 40.91(CH_2_), 29.47(CH_2_).

*N-(3,4-Methylenedioxyphenethyl)-2-(2-bromophenyl)acetamide* (**8c**). In a similar manner, **8c** was obtained in 82.5% yield as a pale yellow solid from ethanol; m.p. 124-126 °C (Lit. [16] m.p. 128-130 °C); ^1^H-NMR: δ 7.56 (1H, d, *J* = 7.8 Hz, Ar-H); 7.33-7.25 (2H, m, Ar-H); 7.19-7.11 (1H, m, Ar-H); 6.65 (1H, d, *J* = 7.9 Hz, Ar-H); 6.55 (1H, d, *J* = 1.6 Hz, Ar-H); 6.48 (1H, dd, *J* = 7.9, 1.6 Hz, Ar-H); 5.91 (2H, s, OCH_2_O); 5.48 (1H, br s, NH); 3.66 (2H, s, CH_2_); 3.42 (2H, apparent q, *J* = 6.7 Hz, CH_2_); 2.65 (2H, apparent t, *J* = 6.7 Hz, CH_2_); ^13^C-NMR: δ 169.44(C), 147.72(C), 146.11(C), 134.80(C), 133.13(CH), 132.35(C), 131.67(CH), 129.09(CH), 127.97(CH), 124.98(C), 121.57(CH), 109.04(CH), 108.30(CH), 100.85(CH_2_), 44.04(CH_2_), 40.87(CH_2_), 35.15(CH_2_).

*1-(2-Bromobenzyl)-3,4-dihydro-6,7-dimethoxyisoquinoline* (**9a**). A solution of **8a** (5.5 g, 14.6 mmol) and phosphorus oxychloride (17.0 g) in benzene (60 mL) was refluxed for 3 h. The excess reagent and solvent were removed under vacuum. The residue was shaken with chloroform (100 mL) and dilute ammonium hydroxide (100 mL). The chloroform layer was washed with water (100 mL), then dried over anhydrous sodium carbonate. Removal of the solvent under vacuum gave dihydroisoquinoline **9a** as a pale yellow solid (3.9 g, 75.8%) from ethyl acetate-hexane; m.p. 95-96 °C (Lit. [15] m.p. 93-95 °C). It was found to be unstable and was immediately used in the next step without further purification. ^1^H-NMR: δ 7.56 (1H, dd, *J* = 7.9, 1.3 Hz, Ar-H); 7.27 (1H, dd, *J* = 7.6, 1.7 Hz, Ar-H); 7.18 (1H, dt, *J* = 7.6, 1.3 Hz, Ar-H); 7.05 (1H, dt, *J* = 7.6, 1.7 Hz, Ar-H); 6.91 (1H, s, Ar-H); 6.67 (1H, s, Ar-H); 4.20 (2H, s, Ar-CH_2_); 3.88 (3H, s, OCH_3_); 3.79 (3H, s, OCH_3_); 3.73 (2H, t, *J* = 7.6 Hz, CH_2_); 2.67 (2H, t, *J* = 7.6 Hz, CH_2_); ^13^C-NMR δ: 165.08(C), 150.76(C), 147.43(C), 137.66(C), 132.78(CH), 131.59(C), 130.21 (CH), 128.18(CH), 127.62(CH), 124.54(C), 121.41(C), 110.25(CH), 109.15(CH), 56.16(OCH_3_), 55.92(OCH_3_), 47.29(CH_2_), 42.53(CH_2_), 25.73(CH_2_). 

*1-(2-Bromobenzyl)-3,4-dihydro-5,6,7-trimethoxyisoquinoline* (**9b**). In a similar manner, **9b** was obtained in almost quantitative yield as a yellow viscous oil. ^1^H-NMR: δ 7.55 (1H, dd, *J* = 7.9, 1.2 Hz, Ar-H); 7.30-7.26 (1H, m, Ar-H); 7.20-7.15 (1H, m, Ar-H); 7.09-7.01 (1H, m, Ar-H); 6.78 (1H, s, H−8); 4.19 (2H, s, Ar−CH_2_); 3.88 (3H, s, OCH_3_); 3.83 (3H, s, OCH_3_); 3.78 (3H, s, OCH_3_); 3.71 (2H, t, *J* = 7.6 Hz, CH_2_); 2.67 (2H, t, *J* = 7.6 Hz, CH_2_); ^13^C-NMR: δ 164.68(C), 151.69(C), 149.88(C), 144.17 (C), 137.63(C), 132.77(CH), 130.23(CH), 128.19(CH), 127.61(CH), 124.52(C), 124.46(C), 124.09(C), 105.63(CH), 60.88(OCH_3_), 60.83(OCH_3_), 56.19(OCH_3_), 47.09 (CH_2_), 42.58(CH_2_), 18.99(CH_2_).

*1-(2-Bromobenzyl)-3,4-dihydro-6,7-methylenedioxyisoquinoline* (**9c**). In a similar manner, **9c** was obtained in 42.1% yield from ethanol as a pale yellow solid; m.p. 122-123 °C (Lit. [16] m.p. 121-123 °C); ^1^H-NMR: δ 7.52 (1H, dd, *J* = 7.8, 1.0 Hz, Ar-H); 7.22-7.12 (2H, m, Ar-H); 7.06- 6.98 (1H, m, Ar-H); 6.89 (1H, s, Ar-H); 6.61 (1H, s, Ar-H); 5.86 (2H, s, OCH_2_O); 4.09 (2H, s, Ar-CH_2_); 3.65 (2H, t, *J* = 7.6 Hz, CH_2_); 2.60 (2H, t, *J* = 7.6 Hz, CH_2_); ^13^C-NMR: δ 164.65(C), 149.08(C), 146.44(C), 137.55(C), 133.38(C), 132.79 (CH), 130.30(CH), 128.14(CH), 127.50(CH), 124.79(C), 122.77(C), 107.98(CH), 106.02(CH), 101.31(CH_2_), 47.09(CH_2_), 42.55(CH_2_), 26.33(CH_2_).

*1-(2-Bromophenyl)-5,6-dihydro-8,9-dimethoxypyrrolo[2,1-a]isoquinoline-2,3-dione* (**10a**). Oxalyl chloride (0.2 mL) was added dropwise to a stirred solution of **9a** (359 mg, 1 mmol), triethylamine (0.3 mL) in chloroform (10 mL) at room temperature. Stirring was continued for 3 h. Chloroform (20 mL) was added and the chloroform layer was washed with 5% hydrochloric acid (4 × 50 mL), water (50 mL), then dried over anhydrous sodium sulfate. Removal of the solvent under vacuum gave a residue which was recrystallized from ethanol to give 10a as red prisms (247.8 mg, 60.0%); m.p. 195-196 °C. (lit. [6] m.p. 176-178 °C); UV λ_max_ (MeOH) nm (log ε): 203 (4.36), 226sh (4.12), 262 (3.76), 322 (3.65). IR (CH_2_Cl_2_-film) ν_max_ cm^-1^: 2937, 2843, 1744, 1699, 1594, 1575, 1515, 1472, 1428, 1398, 1337, 1312, 1291, 1270, 1225, 1187, 1101, 1034, 987, 865, 798, 735, 683; ^1^H-NMR: δ 7.70 (1H, dd, *J* = 8.0, 0.9 Hz, Ar-H); 7.44-7.37 (1H, m, Ar-H); 7.34-7.29 (1H, m, Ar-H); 7.29-7.22 (1H, m, Ar-H); 6.77 (1H, s, Ar-H); 6.66 (1H, s, Ar-H); 3.95 (3H, s, OCH_3_); 3.89 (2H, t, *J* = 6.3 Hz, CH_2_); 3.29 (3H, s, OCH_3_); 3.10 (2H, t, *J* = 6.3 Hz, CH_2_); ^13^C-NMR: δ 181.59(C), 158.50(C), 158.35(C), 153.80(C), 148.14(C), 133.17(CH), 133.09(C), 132.83(CH), 132.41(C), 129.96(CH), 128.06(CH), 125.82(C), 116.68(C), 111.29(CH), 107.80(C), 56.27(OCH_3_), 55.17(OCH_3_), 36.37(CH_2_), 28.37(CH_2_). HRMS (ESI-TOF) calcd for C_20_H_16_BrNO_4_ ([M+H^+^]) = 414.0335, Found 414.0438. 

*1-(2-Bromophenyl)-5,6-dihydro-7,8,9-trimethoxypyrrolo[2,1-a]isoquinoline-2,3-dione* (**10b**). In a similar manner, **10b** was obtained as a deep red solid in 68.2% yield after chromatography over alumina using dichloromethane as eluent; m.p. 69-70 °C; UV (MeOH) λ _max_ nm (log ε): 203 (4.66), 226sh (4.38), 258 (3.95), 332 (3.93); IR (CH_2_Cl_2_-film) ν_max_ cm^-1^: 2939, 2837, 1746, 1702, 1592, 1576, 1467, 1425, 1397, 1342, 1298, 1248, 1182, 1109, 1024, 986, 939, 914, 845, 752; ^1^H-NMR: δ 7.71 (1H, dd, *J* = 8.0, 0.9 Hz, Ar-H); 7.44-7.35 (1H, m, Ar-H); 7.32-7.23 (2H, m, Ar-H); 6.53 (1H, s, Ar-H); 3.94 (3H, s, OCH_3_); 3.88 (3H, s, OCH_3_); 3.93-3.73 (2H, m, CH_2_); 3.27 (3H, s, OCH_3_); 3.20-3.00 (2H, m, CH_2_); ^13^C-NMR: δ 182.05(C), 158.07(C), 152.24(C), 150.56(C), 147.02(C), 133.21(CH), 132.70(CH), 132.25(C), 130.03(CH), 128.08(CH), 125.77(C), 125.48(C), 119.38(C), 108.64(C), 108.53(CH), 105.68(C), 61.12(OCH_3_), 61.08(OCH_3_), 55.21(OCH_3_), 36.19(CH_2_), 21.67(CH_2_). HRMS (ESI-TOF) calcd for C_21_H_18_BrNO_5_ ([M+H^+^]) = 444.0441, Found 444.0519.

*1-(2-Bromophenyl)-5,6-dihydro-8,9-methylenedioxypyrrolo[2,1-a]isoquinoline-2,3-dione* (**10c**). In a similar manner, **10c** was obtained in 47.7% yield from ethanol as a deep red prisms; m.p. 226-227 °C; UV (MeOH) λ _max_ nm (log ε): 203 (4.64), 236sh (4.18), 261sh (3.88), 284 (3.77), 320 (3.67), 388 (3.64); IR (CH_2_Cl_2_-film) ν_max_ cm^-1^: 3056, 2906, 1744, 1698, 1608, 1568, 1505, 1467, 1403, 1378, 1338, 1316, 1286, 1249, 1181, 1036, 938, 868, 748, 736; ^1^H-NMR: δ 7.71-7.66 (1H, m, Ar-H); 7.42-7.36 (1H, m, Ar-H); 7.31-7.23 (2H, m, Ar-H); 6.79 (1H, s, Ar-H); 6.56 (1H, s, Ar-H); 6.00 (2H, AB q, *J* = 1.1 Hz, OCH_2_O); 3.94-3.77 (2H, m, CH_2_); 3.07 (2H, t, *J* = 6.3 Hz, CH_2_); ^13^C-NMR: δ 181.94(C), 158.10(C), 157.94(C), 152.47(C), 147.37(C), 135.17(C), 133.43(CH), 132.43(CH), 131.68(C), 130.13(CH), 128.14(CH), 125.35(C), 118.23(C), 109.28(CH), 108.50(CH), 108.23(C), 102.25(CH_2_), 36.20(CH_2_), 29.24(CH_2_). HRMS (ESI-TOF) calcd for C_19_H_12_BrNO_4_ ([M+H^+^]) = 398.0022, Found 397.9895.

*Telisatin A* (**1**). A solution of 1,1ʹ-azobis(cyclohexanecarbonitrile) (245.0 mg, 1.0 mmol) and tributyltin hydride (1.2 g, 4.0 mmol) in toluene (20 mL) was added dropwise in four equal portions over 3 h to a refluxing solution of **10a** (413.0 mg, 1.0 mmol) in toluene (20 mL) and the resulting mixture was then refluxed for another 8 h. The solvent was then removed under vacuum and the residue was dissolved in acetonitrile (40 mL) and washed with hexane (2 × 30 mL), then dried over anhydrous sodium sulfate. Removal of the solvent gave a brown viscous oil (0.4 g) which was recrystallized with ethanol to give telisatin A (**1**) as red prisms (109.9 mg, 33.0%); m.p. 234-235 °C (Lit. [1] m.p. 238-239 °C); UV (MeOH) λ _max_ nm (log ε): 207 (4.03), 257 (4.26), 284sh (3.60), 322 (3.70), 336 (3.80), 352sh (3.56); IR (CH_2_Cl_2_-film) ν_max_ cm^-1^: 2925, 1748, 1701, 1605, 1584, 1531, 1462, 1423, 1386, 1306, 1261, 1195, 1149, 1131, 1112, 1037, 969, 924, 802, 759. ^1^H-NMR δ: 9.41 (1H, br d, *J* = 8.5 Hz, H−11); 8.63 (1H, dd, *J* = 8.0, 1.5 Hz, H−8); 7.67-7.60 (1H, m, H−9); 7.54-7.46 (1H, m*,* H−10); 7.17 (1H, s, H−3); 4.10 (3H, s, OCH_3_); 3.97 (2H, t, *J* = 6.5 Hz, CH_2_); 3.95 (3H, s, OCH_3_); 3.35 (2H, t, *J* = 6.5 Hz, CH_2_); ^13^C-NMR: δ 179.98(C), 160.34(C), 157.15(C), 153.36(C), 146.65(C), 130.75(C), 129.35(C), 129.21(CH), 128.33(CH), 127.56(C), 125.87(C), 125.62(CH), 123.76(CH), 112.29(CH), 112.18(C), 103.17(C), 59.99(OCH_3_), 56.62(OCH_3_), 36.53(CH_2_), 27.68 (CH_2_). HRMS (ESI-TOF) calcd for C_20_H_15_NO_4_ ([M+H^+^]) = 334.1074, Found 334.1125.

*Telisatin B* (**2**). In a similar manner, telisatin B (**2**) was obtained as deep red prisms (30.0%) from ethanol; m.p. 218-219 °C (Lit.[1] m.p. 221-222 °C); UV (MeOH) λ _max_ nm (log ε): 203 (4.33), 223sh (4.26), 257 (4.59), 318sh (3.99), 329 (4.07); IR (CH_2_Cl_2_-film) ν_max_ cm^-1^: 2942, 2864, 1749, 1716, 1702, 1619, 1607, 1579, 1527, 1515, 1452, 1406, 1389, 1323, 1146, 1125, 1071, 1033, 973, 814, 757; ^1^H-NMR: δ 9.37 (1H, br d, *J* = 8.5 Hz, H−11), 8.60 (1H, dd, *J* = 8.0, 1.3 Hz, H−8), 7.64-7.56 (1H, m, H−9), 7.55-7.45 (1H, m, H−10), 4.14 (3H, s, OCH_3_), 4.03 (3H, s, OCH_3_), 4.01 (3H, s, OCH_3_), 3.91 (2H, t, *J* = 6.5 Hz, CH_2_), 3.33 (2H, t, *J* = 6.5 Hz, CH_2_); ^13^C-NMR: δ 180.69(C), 159.86(C), 152.58(C), 152.16(C), 152.03(C), 150.16(C), 128.71(CH), 127.53(CH), 126.63(C), 126.38(C), 126.11(C), 125.78(CH), 123.66(CH), 121.18(C), 114.14(C), 104.17(C), 61.47(OCH_3_), 61.30(OCH_3_), 60.48 (OCH_3_), 36.14(CH_2_), 21.21(CH_2_); HRMS (ESI-TOF) calcd for C_21_H_17_NO_5_ ([M+H^+^]) = 364.1179, Found 364.1231.

*Lettowianthine* (**3**). In a similar manner, lettowianthine (**3**) was obtained as red prisms (34.0%); m.p. 294-295 °C (dec.) (Lit. [2] m.p. 314-317 °C (dec.); Lit.[4] m.p. 265-267 °C); UV (MeOH) λ _max_ nm (log ε): 203 (4.37), 212sh (4.29), 247sh (4.07), 257sh (4.04), 287 (3.64), 335 (3.51), 353 (3.28). IR (CH_2_Cl_2_-film) ν_max_ cm^-1^: 2923, 2093, 1737, 1695, 1622, 1610, 1581, 1530, 1506, 1450, 1417, 1301, 1253, 1222, 1176, 1151, 1122, 1050, 927, 867, 749; ^1^H-NMR: δ 8.80 (1H, br d, *J* = 8.6 Hz, H−11), 8.55 (1H, br d, *J* = 7.6 Hz, H−8), 7.61 (1H, t, *J* = 6.9 Hz, H−9), 7.48 (1H, t, *J* = 7.2 Hz, H−10), 7.09 (1H, s, H−3), 6.35 (2H, s, OCH_2_O), 3.93 (2H, t, *J* = 6.2 Hz, CH_2_), 3.29 (2H, t, *J* = 6.2 Hz, CH_2_); ^13^C-NMR δ: 179.92(C), 160.33(C), 153.68(C), 151.55(C), 143.08(C), 129.61(C), 129.33(CH), 127.62 (CH), 126.83(C), 125.35(CH), 124.45(C), 123.60(CH), 119.90(C), 112.46(C), 109.23(CH), 103.10 (C), 102.35(CH_2_), 36.66(CH_2_), 27.51(CH_2_); HRMS (ESI-TOF) calcd for C_19_H_11_NO_4_ ([M+H^+^]) = 318.0761, Found 318.0666.
